# Cuspal Displacement Induced by Bulk Fill Resin Composite Polymerization: Biomechanical Evaluation Using Fiber Bragg Grating Sensors

**DOI:** 10.1155/2016/7134283

**Published:** 2016-04-12

**Authors:** Alexandra Vinagre, João Ramos, Sofia Alves, Ana Messias, Nélia Alberto, Rogério Nogueira

**Affiliations:** ^1^Faculty of Medicine, University of Coimbra, Avenida Bissaya Barreto, Blocos de Celas, 3000-075 Coimbra, Portugal; ^2^Instituto de Telecomunicações (IT), Campus Universitário de Santiago, 3810-193 Aveiro, Portugal

## Abstract

Polymerization shrinkage is a major concern to the clinical success of direct composite resin restorations. The aim of this study was to compare the effect of polymerization shrinkage strain of two resin composites on cuspal movement based on the use of fiber Bragg grating (FBG) sensors. Twenty standardized Class II cavities prepared in upper third molars were allocated into two groups (*n* = 10). Restorations involved the bulk fill placement of conventional microhybrid resin composite (Esthet•X® HD, Dentsply DeTrey) (Group 1) or flowable “low-shrinkage” resin composite (SDR*™*, Dentsply DeTrey) (Group 2). Two FBG sensors were used per restoration for real-time measurement of cuspal linear deformation and temperature variation. Group comparisons were determined using ANCOVA (*α* = 0.05) considering temperature as the covariate. A statistically significant correlation between cuspal deflection, time, and material was observed (*p* < 0.01). Cuspal deflection reached 8.8 *μ*m (0.23%) and 7.8 *μ*m (0.20%) in Groups 1 and 2, respectively. When used with bulk fill technique, flowable resin composite SDR*™* induced significantly less cuspal deflection than the conventional resin composite Esthet•X® HD (*p* = 0.015) and presented a smoother curve slope during the polymerization. FBG sensors appear to be a valid tool for accurate real-time monitoring of cuspal deformation.

## 1. Introduction

Volumetric shrinkage remains a major drawback to the clinical performance of the resin composite restorations. Shrinkage leads to deformation of the resin composites and generates stress due to the confinement of the resin to the cavity walls generated by the bonding procedure. This shrinkage stress is transferred to the tooth and may lead to cuspal deflection or enamel microcracks, whereas stress at the tooth-composite interface increases the likelihood of interfacial adhesive failures [1].

Cuspal deflection occurs due to the interaction between the polymerization shrinkage stress of the resin composite, the adhesive interface, and the compliance of the cavity wall [2]. Compliance is defined as the change in dimension per unit of force applied or generated, being essentially the inverse of stiffness [1]. Several studies have described it as a valuable method to assess the effects of polymerization shrinkage stress [3–7] and dimensional changes have been reported to range from 4 to 25 *μ*m [4, 6, 8, 9]. The amplitude of this inward cuspal movement can depend on several factors, namely, the size and configuration of the cavity [2, 3, 10]; the properties of the resin composite [2, 4, 5, 9]; the bonding system [3, 5]; the hydration condition of the teeth [2]; and the experimental conditions [4]. Even though different model designs have been used for cusp deflection assessment, such as glass rods, aluminum blocks, or tooth structure, all inherently present with distinct compliance behaviors [4, 11, 12]. In order to overcome this limitation, system compliance similar to that of teeth is necessary to accurately detect stress [4, 11, 12]. Considering substrate structural deformability, both C-factor and resin composite volume seem to have an impact on the substrate compliance. When the substrate is only slightly deformable, the increase of the stress correlates better with the C-factor but if the compliance is higher, the resin composite volume would correlate better with stress development [10]. These findings demand careful data interpretation across studies concerning different methodologies for cuspal deflection assessment.

Additionally, the development of inward cuspal deflection can also be related to the strategies employed for managing shrinkage stress of resin composites [1]. These clinical approaches to reduce polymerization shrinkage include incremental placement techniques [2, 4, 11], the use of low-modulus intermediate liner materials as stress absorbers [4, 7], and modification of the light application methods to reduce curing speed [13]. Also, factors related to resin composite formulations like changes in filler amount, shape or surface treatment, variations in monomer structure or chemistry, and modification of polymerization resin kinetics have been more recently introduced aiming to reduce the polymerization shrinkage [1, 4, 5, 13]. All these strategies encompass a new class of resin composites known as “low-shrinkage resin based composites” that are generally allowed to be placed in a bulk fill mode due to the increased depth of cure, probably related to higher translucency [14]. Bulk filling techniques are undoubtedly more user friendly than the necessary meticulous incremental layering techniques advocated for conventional resin based composites (RBCs) [8], which justifies the growing interest in these so-called “low-shrinkage” RBCs and raises the need for exhaustive studies to clarify their potentialities [5, 8, 14–16].

Many methods have been used to evaluate cuspal deflection, involving technologies that go from linear variable differential transformers (LVDT) [2, 4, 11], strain gauges [9], profilometry [3], or twin channel deflection measuring gauge [6–8], among others. Fiber Bragg grating (FBG) sensors can be used to perform real-time local temperature and strain measurements [17–20]. Fiber optical sensors have the advantage of presenting immunity to electromagnetic interference [21], small dimensions [17–20], high resolution and sensibility, chemical inertness [17–19], biocompatibility [17], long-term stability [20], multiplexing capability, possibility to be embedded in different structures [17, 22], and ability to perform remote measurements [21].

The aim of this study was to compare the cuspal displacement induced by the polymerization shrinkage of a bulk fill resin composite (SDR*™*) and a conventional microhybrid resin composite (Esthet•X® HD) using fiber Bragg grating (FBG) sensors. The null hypothesis stated that there are no significant differences in cuspal displacement generated by the two resin composites.

## 2. Materials and Methods

### 2.1. Tooth Selection and Cavity Preparation

Twenty caries free, intact, and freshly extracted human upper third molars were collected after the patient's informed consent, as approved by the Ethical Committee of the Faculty of Medicine of Coimbra, Portugal (CE-001/2013). The teeth were cleaned and visually inspected to guarantee absence of hypoplastic defects, fractures, or cracks. Teeth were then stored at room temperature in a 10% buffered formalin solution (pH 7.0) for up to 3 months after extraction.

To standardize the dimensions of the molars, the teeth were selected based on the maximum buccal-palatal width (BPW), varying between 9.5 mm and 10.6 mm, and on the mesiodistal distance, varying between 8.1 mm and 9.7 mm, measured with a digital micrometer gauge (105–156, Mitutoyo, IL, Chicago, USA). Teeth were then randomly distributed into two groups (*n* = 10) ensuring a variance of the mean BPW between groups lower than 5%.

**Figure 1:**
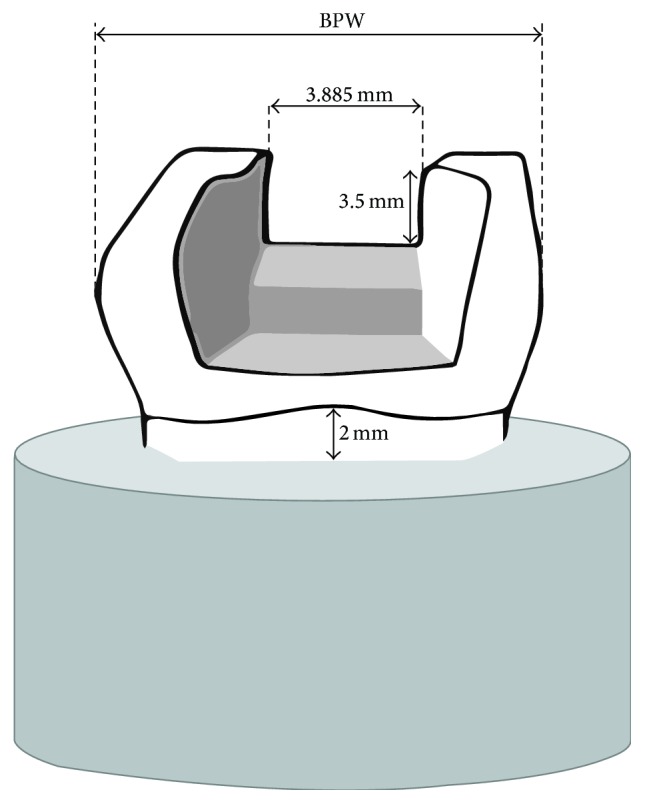
Schematic illustration of the shape and dimensions of the cavity (adapted from Palin et al. 2005 [5]).

Each tooth was embedded in self-polymerizing acrylic resin (Orthocryl®, Dentaurum Ispringen, Germany) 2 mm apical from the cement-enamel junction (CEJ), with the long axis vertically oriented. Standardized large mesio-occluso-distal (MOD) cavities were prepared in each molar using a tungsten carbide round-ended bur in a high-speed handpiece (G848-314-031-10-ML, Diamond FG, Colténe/Whaledent AG, Switzerland) with copious water irrigation followed by a cone shaped bur of large diameter (980.040, set 4273, Komet®, Germany) mounted in a low-speed handpiece with water coolant indicated for inlay cavity preparations. The cusps were minimally frayed and all internal angles rounded. The width of the proximal boxes was approximately two-thirds the BPW (Figure 1). The occlusal isthmus was prepared approximately to half of the BPW (3.885 mm). The cavity depth was standardized to 3.5 mm from the tip of cusps at the occlusal isthmus and 1 mm above the CEJ at the cervical aspect of the proximal boxes (adapted from Palin et al. 2005 [5]).

After cavity preparation, adhesive procedures were performed using a two-step etch and rinse adhesive system (Prime&Bond®NT*™*, Dentsply DeTrey, Konstanz, Germany) according to manufacturer instructions followed by a 10-second light-curing exposure with a LED light-curing unit (Bluephase®, Ivoclar Vivadent, Liechenstein) in its “low mode” program emitting 650 mW/cm^2^ (Table 1). A second layer of adhesive was then applied and cured in similar way. Afterwards, teeth were passively surrounded by a band of polytetrafluoroethylene (Teflon®, DuPont, Wilmington, DE, USA) to prevent resin overflow during restorative procedures with either the bulk fill resin composite SDR*™* or the microhybrid Esthet•X® HD (Dentsply DeTrey, Konstanz, Germany) used as control (Table 1).

### 2.2. Measurement of Cuspal Deflection with FBGs

A FBG is a periodic modulation of the refractive index along the core of an optical fiber. This modulation operates as a highly selective wavelength filter. When a FBG is illuminated by a broadband light source, only wavelengths that satisfy the Bragg condition are reflected, while all the others are transmitted.

The Bragg condition is given by the following:

(1) $$\begin{array}{c} \lambda_{B}=2\Lambda n_{eff}, \end{array}$$

where *λ*_*B*_ is the Bragg wavelength, Λ is the periodic modulation of the refractive index, and *n*_eff_ is the effective refractive index of the fiber core.

The effective refractive index, as well as the periodic spacing between the grating planes, will be affected by changes in strain and/or temperature which will modify the center wavelength of light back reflected from the Bragg grating. Using the first equation, the shift in the Bragg grating center wavelength due to strain and temperature changes is given by the following:

(2) ΔλBΔλB,l+ΔλB,T=2Λ∂n∂l+n∂Λ∂lΔl+2Λ∂n∂T+n∂Λ∂TΔT=SlΔl+STΔT,

where Δ*λ*_*B*,*l*_ is the strain induced wavelength shift and Δ*λ*_*B*,*T*_ is the thermal effect on the same parameter. *S*_*l*_ and *S*_*T*_ represent the strain and temperature sensitivity coefficients of the FBG sensors [23]. For this work, they were previously determined and 0.00118 nm/*με* and 0.0089 nm/°C were obtained, respectively.

In the current study, gratings with 1 mm length were inscribed onto photosensitive optical fiber (FiberCore PS 1250/1500) with a UV light (248 nm) from a KrF excimer laser, using the phase mask technique.

**Figure 2:**
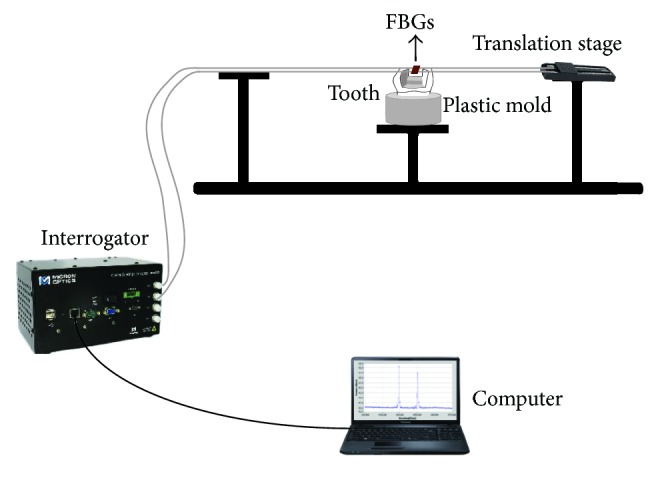
Schematic overview of the experimental setup used to measure the setting cuspal deformation and the temperature variation.

One drawback of the FBG based sensors is their cross-sensitivity to both strain and temperature. For that, in this study two FBGs were used to measure the setting cuspal deformation and the temperature variation. One of them, sensitive to strain and temperature variations, was placed perpendicular to the buccal cusp. This grating was previously tensioned (about 500 *με*), allowing the FBG sensor to detect not only the increasing of the distance between the cusps but also its approximation. For this, one side of the fiber was glued (Loctite®, Henkel, Germany, and cyanoacrylate accelerator, Pekecho®, Spain) to a controllable translation stage and the other one to the cusp farthest from it. After the glue drying time (15 min), the fiber was tensioned and bonded to the other cusp (nearest of the controllable translation stage). A new waiting period of 15 min was performed. The second FBG was placed parallel to the first one, but not bonded nor pretensioned, being only sensitive to temperature variations. The gratings' wavelength was measured using a sm 125–500 interrogation system (Micron Optics Inc., Atlanta, USA) with a measurement range of 1510–1590 nm, wavelength resolution of 1 pm, and an acquisition frequency of 2 Hz.  Figure 2 shows a schematic representation of the experimental apparatus.

After the setup, the cavities were gently bulk filled with resin composite according to two groups (SDR*™* or Esthet•X® HD) and the same LED light-curing unit was used for polymerization in the soft-start mode, running at 650 mW/cm^2^ for the first 5 seconds and at 1200 mW/cm^2^ in the following period. Light-curing tip was placed 1 mm above the samples, with an incidence angle near 90°. The samples were initially irradiated for 30 seconds and after a 5-minute break a second light-curing period of 30 seconds was applied. In all tests, data were continuously acquired for 10 minutes from the beginning of the polymerization.

The experiment was repeated alternately for each resin, performing a total number of ten samples per group. Beside these tests, the thermal variation caused by the light-curing unit was further investigated using a similar experimental setup, but applying only the temperature sensitive sensor without filling the cavity with resin composite.

All the experiments were performed under controlled room temperature conditions (21°C).

### 2.3. Statistical Analysis

Statistical analysis was performed using SPSS 20.0® (SPSS Inc., Chicago, IL, EUA). Cuspal deflection variation between groups was determined with ANCOVA, considering temperature as a covariate. Repeated measures ANOVA considering Greenhouse correction was used to analyze cuspal deflection variation within time for each group. Significance level was set at *α* = 0.05.

## 3. Results

**Figure 3:**
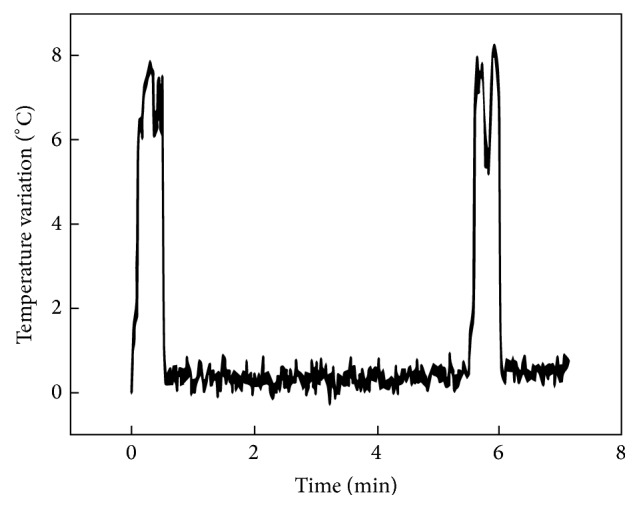
Temperature variation induced by the LED light-curing unit.

Temperature rise induced by the irradiation with the LED light-curing unit is represented in Figure 3. In the soft-start mode, the first and second irradiation periods reached a temperature rise of 7.9°C and 8.3°C, respectively.

**Figure 4:**
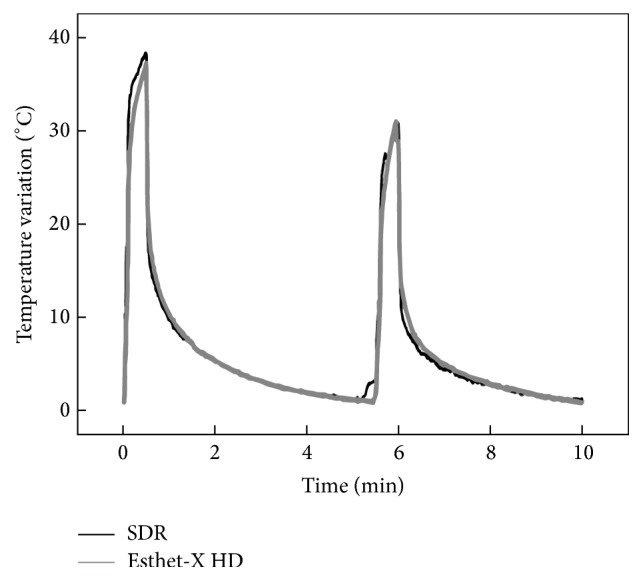
Average temperature variation obtained during the light curing of the restorations with the SDR*™* and Esthet•X® HD resins.

The mean temperature rise registered from all tests during light curing of the restorations is shown in Figure 4. For Esthet•X® HD, the temperature increased 36.7°C and 28.9°C at the final of the first and second irradiation period, respectively. In the case of the SDR*™*, a temperature variation of 38.2°C and 30.8°C was obtained for the same periods.

**Figure 5:**
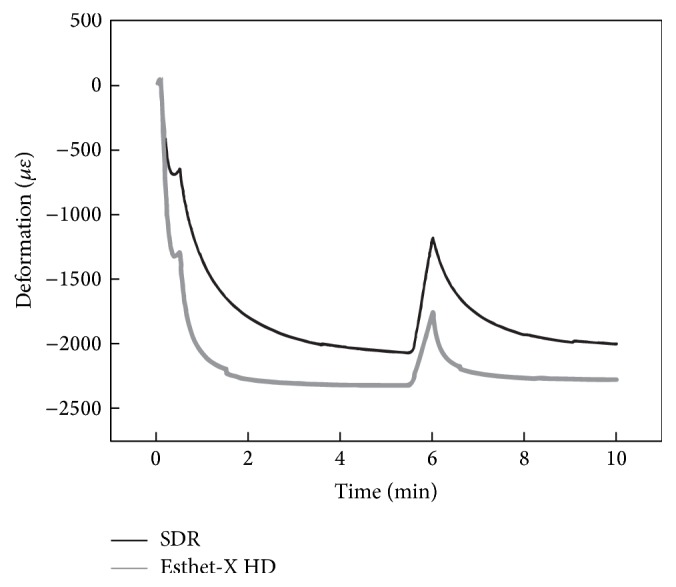
Average cuspal deformation induced by SDR*™* and Esthet•X® HD resins during the 10 minutes of real-time monitoring.

The average cuspal deformation, resulting from the ten tests of each resin, during the 10 minutes of monitoring is represented in Figure 5. The curves were obtained by subtracting the effect of the temperature, obtained with the grating inscribed in the fiber that was not bonded, from the measurements accomplished by the other sensor, which is sensitive to both temperature and strain variations.

Descriptive statistics of cuspal deflection in each period is shown in Table 2. Between-subjects analysis of covariance (ANCOVA) is summarized in Table 3.

Over the time of polymerization, the two resin composites presented a significant variation in material behavior (*F*(1.42,24.19) = 245.37, *p* < 0.01, partial *η* ^2^ = 0.935), as expressed in Figure 5. After the first polymerization period, both groups present statistically significant cuspal inwards deformation in relation to any other measurement (*p* < 0.01 for all comparisons between 0.5 minutes and 5.5, 6, and 10 minutes within each group). A similar behavior was found after the second polymerization period (6 to 10 minutes) but with lower deformation (*p* < 0.01). Only SDR presented no differences in cuspal deformation after the postpolymerization clearance periods (*p* = 0.051 for the 5.5- and 10-minute comparison).

When light curing started, cuspal deformation experienced a slight expansion curve for both materials evaluated. This peak reached the maximum value at 0.05 minutes for both resin composites. Esthet•X® achieved in average 27.1 *με* and SDR*™* 91.8 *με*. Thereafter, curves decreased (Figure 5). At 30 seconds, the mean values of cuspal deflection were −1299.5 *με* and −646.3 *με* for Esthet•X® HD and SDR*™*, respectively (Figure 5 and Table 2). ANCOVA analysis considering temperature as the covariate revealed a statistically significant difference in cuspal deflection between the two resin composite resins at 30 seconds of polymerization (*F*(1,17) = 52.69, *p* < 0.01, partial *η* ^2^ = 0.756). *t*-test for independent samples equality of means revealed deformation values statistically significantly higher in Esthet•X® HD compared to SDR*™* (Table 3).

In the mean time until the second polymerization (0.5 to 5.5 minutes), Esthet•X® HD curve deflection decreased continuously until 2 minutes pass. From this moment, values remained constant until the terminus of this light-curing free period, reaching a mean of −2296.9 *με* at 5.5 minutes. The SDR*™* curve declines at a slower rate. At 5.5 minutes, samples restored with SDR*™* presented a cuspal deflection of −2074.1 *με* (Figure 5 and Table 2). There was a statistically significant difference in cuspal deflection between two groups at this point of the curing protocol (*p* = 0.016) (Table 3).

During the second polymerization period, a new expansion peak occurred for both resin composites. After that, at 6 minutes, Esthet•X® HD curve presented −1751.4 *με* of cuspal deflection and SDR*™*  −1166.3 *με* (Table 2 and Figure 5). According to that, between the beginning and the ending of the second polymerization period, cusps expanded approximately 560.1 *με* in samples restored with Esthet•X® HD and 907.8 *με* in SDR*™* samples. Mixed ANOVA considering Greenhouse correction revealed a statistically significant difference in cuspal deflection between the two resin composites at 6 minutes (*F*(1,10) = 11.81, *p* = 0.006, partial *η* ^2^ = 0.542) (Table 3), meaning that cuspal deflection of Esthet•X® HD at this time point was significantly higher than SDR*™*.

In the period from 6 to 10 minutes, both deformation curves decreased and for the last evaluated point the values remained constant (Figure 5). The average results obtained with Esthet•X® HD and SDR*™*, at 10 minutes, were −2277.2 *με* and −2001.2 *με*, respectively (Table 2). There was a statistically significant difference in cuspal deflection between the two materials at 10 minutes (*p* = 0.015) (Table 3).

In order to determine the shortening distance between the cusps (cuspal deflection), deformation expressed in microstrain (*με*) was converted to micrometers (*μ*m), according to the following equation:

(3) Cuspal deflectionμm=Deformationμε∗Initial distant between cuspids3885 μm1∗106.

The average results obtained in micrometers (*μ*m) for the more relevant periods of 0.5 and 10 min were 5.0 *μ*m (0.13%) and 8.8 *μ*m (0.23%) in the case of the Esthet•X® HD and 2.5 *μ*m (0.06%) and 7.8 *μ*m (0.20%) for the SDR*™* resin, respectively.

## 4. Discussion

Despite numerous studies published, the highly complex phenomenon of polymerization shrinkage that develops in polymeric dental restorative materials is not yet fully understood and remains a significant clinical concern. This phenomenon becomes even more complex when the composite is bonded into cavities of variable configurations. For resin composites bonded to enamel and dentin, polymerization shrinkage is constrained and polymerization stress development becomes more complex due to the generation of interfacial stresses usually unevenly distributed along the cavity walls and the bonded composite surfaces [10, 12].

The present study measured the tooth deformation instead of shrinkage stresses, which can be assumed as an indirect indicator associated with internal stress [3, 4]. Regrettably, the methods most commonly used for cuspal deflection monitoring depend primarily on measuring the difference between precuring and postcuring values, but they do not provide detailed data regarding how this phenomenon occurs in a real-time process [9]. In fact, the methods of measurement of cusp deflection during composite restoration have been reported to produce considerably varying results. When applying contact methods, the use of reproducible reference points on the cusps seems to be critical to avoid erroneous results between samples [5]. With FBG sensors this point is not an issue since the measurement of dimensional changes is made by the Bragg sensor, which is not directly attached to the tooth structure. Therefore, it can be expected that under identical experimental conditions, cusp deflection measurements for the same restoration protocol can result in different data according to the experimental device used and the inherent tooth compliance. Caution is needed when comparing results across studies as mean cuspal deflections of up to 50 *μ*m were recorded using a wide range of techniques [2–4, 6–9, 11].

Human molars were used to assess cuspal deflection using FBG sensors. A relatively high variation among teeth dimensions in experimental studies may affect the outcomes, impairing the comparison between studies with the same purpose. Despite the careful attempts to achieve standardization of cavity preparation, some inevitable discrepancies may be present when natural teeth are used. In this study, molar teeth were selectively allocated in order to promote a maximum difference of 5% in BPW between the groups. Despite tooth standardization, the dispersion values (SD) of each group reported high variability among samples, which could be due to the employment of natural teeth with nonhomogenous morphological and structural characteristics as previously described for other experimental models using natural teeth. In fact, it was not possible to determine the residual thickness of both dentin and enamel, which in conjunction with slight differences in the amount of the resin used to fill the tooth could compromise the compliance behavior and consequently induce different cuspal displacements. Notwithstanding this, molar teeth choice together with the large Class II MOD cavity design used in the present study has the advantage of wider surface area for fiber bonding, contrarily to premolars mostly used in other studies [2, 6, 7] while providing an in vitro simulation of some clinical situations of weakened remaining tooth structure, favorable to cuspal deflection during restorative procedures. Large deflections for MODs cavities can be explained by the loss of tooth rigidity when the marginal ridges are removed [3]. Additionally, the cusps that remain after an MOD cavity preparation were reported to act as a cantilever beam under occlusal load, which increases with cavity depth, while the prepared cavity floor acts as a fulcrum for cusp bending. Biomechanical principles refer to the fact that deflection is proportional to the cubed power of the length and to the inverse of the thickness of the cantilever cusp cubed [7, 8].

FBG sensors methodology allowed a real-time monitoring of the deformations occurring during resin composite curing as well as the thermal behavior during this procedure [17, 20]. For cavity preparations restored with resin composites under shrinkage, loadings and displacements will occur in multiple directions. While knowing that shrinking composite develops a triaxial stress state, as reported with finite element analysis [10, 12], our measurements registered only the forces developing uniaxially in the long axis of each tooth, expressed as a single value. Nevertheless, the use of a standardized tooth cavity allowed a well-balanced homogenization of compliance, cavity configuration, and composite volume, which have been found to be the most critical variables related to stress development in a clinical situation [10].

The present study compared the marketed low-shrinkage flowable RBC SDR*™* with a conventional microhybrid RBC Esthet•X® HD assessing their performance in bulk fill adhesive MOD molar restorations by measuring cuspal deformation with FBG sensors during a two-step curing protocol in order to deliver a reliable energy density to the restoration. Although conventional microhybrid RBCs have restricted indications for bulk fill placement technique and SDR*™* application advocates the use of a microhybrid resin composite for the final covering layer, the purpose was to equalize the restorative protocol in order to isolate and evaluate the individual biomechanical behavior of both materials in similar one-increment situations related to the potential advantage evocated for the “low-shrinkage” one. Significant differences were found between the two materials; therefore the null hypothesis was rejected.

The greatest difference was detected at the end of the first 30 seconds curing period. At this point, Esthet•X® HD induced significantly more deformation (meaning cuspal deflection) than SDR*™*, which could enhance faster and higher stress development at the tooth/restoration adhesive interface. At the end of the subsequent five-minute pause period both RBCs reached higher shrinkage values, although the polymerization kinetics of SDR*™* seems to develop more gradually than that achieved by Esthet•X® HD. This may be related to the functionality of the polymerization modulator incorporated in SDR*™* resin matrix. In theory, when this modulator interacts with the photoinitiator (camphorquinone) the polymerization kinetics can be controlled by delaying the gel point, by slowing the rate of polymerization and elastic modulus development, and by reducing polymer cross-linking and, consequently, shrinkage stress [16, 24].

Cuspal deformation curves showed three expansion peaks during the experimental curing period for both resin composites. As soon as the first light irradiation period started, a discrete expansion and transitory peak were detected. However, at the beginning of the second curing light exposure, an expressive and prolonged expansion peak could be observed during all the 30-second irradiation time. These events can be interpreted as the thermal expansion effect caused either by the heat from the curing light or by the exothermic nature of the free radical polymerization of dimethacrylate monomers, as pointed out by other authors [4, 19, 20]. When polymerization shrinkage exceeds thermal expansion in the first expansion peak, fast overall material shrinking takes place, evidenced by a sudden increase contraction strain [19, 20]. In opposite, the persistent expansion peak observed along the second irradiation period occurs when a considerable cross-linking of the monomer has already been achieved, meaning that the thermal effect has greater relative influence on the dimensional behavior of both resin composites. Additionally, the inherent temperature rise can be implied in the resin composite glass transition temperature attainment, at which the polymer goes from the glassy to the rubbery state [25, 26]. If this occurs, a significant increase in polymer chain mobility is expected, favoring additional cross-linking and stress relief [27]. This expansion was significantly more pronounced for samples restored with SDR*™* than Esthet•X® HD. This can be further explained by the lower filler content exhibited by SDR*™*, as an inverse linear relationship between coefficient of linear thermal expansion and the filler volume fraction of the resin composite has been observed by different researchers [25]. Another intermediate and discrete expansion peak was obtained immediately few seconds before the end of the first irradiation period. Possibly, a cumulative thermal effect induced by the high power density emitted by the curing unit at this point leads to the development of a new expansion phase. These thermal expansions can be considered internal constraints that will be added to the total amplitude internal stress [1].

In the last measurement (10 minutes), the mean total cuspal deflection was 8.8 *μ*m (0.23%) and 7.8 *μ*m (0.20%) for the maxillary molar teeth restored with Esthet•X® HD and SDR*™* resins, respectively. SDR*™* presented significantly less final cuspal deflection than Esthet•X® HD. Indeed, in the few studies available concerning SDR*™*, polymerization stress was reported to be considerably lower than that of conventional flowable resin composites, being comparable to other marketed low shrinking resin composites [8, 16] and marginal integrity appeared as good as that obtained with a conventionally layered resin composite [8]. Previous studies showed volumetric polymerization shrinkage around 3% for Esthet•X® [28] and around 3.1% for SDR*™* [15]. Differences between those findings and the results of the present work could be due to the fact that shrinkage stress development is not exclusively associated with the volumetric shrinkage behavior. Moorthy et al. [8] showed that two bulk fill flowable RBC bases (SDR*™* and x-tra base) have significantly reduced cuspal deflection during light irradiation when compared to a conventional RBC (GrandioSO), reporting a total mean cuspal deflection of 4.63 *μ*m (1.19), 4.73 *μ*m (0.99), and 11.26 *μ*m (2.56) for SDR*™*, x-tra base, and GrandioSO, respectively. Other recent studies conducted by Tauböck et al. [24] reported that SDR*™* generated significantly lower shrinkage forces compared with the microhybrid Esthet•X® HD when irradiation was performed at continuous high irradiance, even though axial polymerization shrinkage of the SDR composite exceeded that of the microhybrid. Nevertheless, the authors revealed no benefit of SDR regarding shrinkage force generation when modulated curing protocols with low initial irradiance were applied, such as in the soft-start mode, arguing a low responsiveness of SDR to modulated photoactivation due to the predominant effect of the polymerization modulator on reaction kinetics and stress development. In the present study, to control shrinkage-induced tensions, both resin composites were polymerized using a soft-start curing mode, with the expectation that this approach could reduce cuspal movement and improvement of restoration interfacial integrity. One can speculate that if a continuous high level curing irradiance had been used, higher cuspal displacement could have taken place, particularly in the microhybrid Esthet•X® HD samples.

Some drawbacks can be pointed out to the methodology employed in this study concerning FBG sensors. Cross-sensitivity to both strain and temperature requires specific techniques to compensate for the thermal influence, which was dealt with as an additional Bragg grating. The information obtained is wavelength encoded and extracting real information from the wavelength shift involves the development of particular applications. Other disadvantages consist on the inherent fiber fragility, which makes the manipulation and the bonding of the fiber to the teeth difficult [17]. Several authors have developed in vitro simulation models to determine the shrinkage stress or cuspal deflection of RBC materials. However, the limitation of some of those in vitro experiments is related to the simplicity of the model designs, using frequently, parallel walls that ignore nonaligned stress development linked to more complex cavity geometries and to the differential compliance of the testing systems, which significantly influences stress development, particularly depending on C-factor and resin composite volume [10]. Also, they do not allow the acquisition of information in a continuous and real-time mode [7, 29, 30], which was overcome by the methodology used in the present study. Another relevant advantage of the FBG is the high resolution obtained in the cuspal displacement monitoring. Since the wavelength resolution of the interrogation system is 1 pm, a variation of 0.85 *με* can be noticed. Considering the initial distance between the cusps of 3885 *μ*m, it corresponds to an absolute resolution of 0.003 *μ*m. During the last few years, research work devoted to studying and exploring the potential application of fiber optic technology in biomedicine has increased significantly [20].

To the extent of the knowledge of the authors concerning the scientific published literature, this is the first known study to measure tooth cuspal deflection with fiber Bragg grating sensors. Fiber Bragg sensors can be extensively applied in future studies, comparing different protocols or clinical modified variables useful for shrinkage stress management in clinical practice.

## 5. Conclusion

Within the conditions of this research protocol, SDR*™* polymerization kinetics induced less stress to dental structure than Esthet•X® HD, as the mean cuspal deflection values were statistically different between the two resin composites.

Despite the limitations of this in vitro study the optical FBG sensors seem to be a suitable measurement method to evaluate tooth dimensional changes related to cuspal deflection induced by resin composite polymerization shrinkage, with some advantages over other techniques, namely, continuous real-time assessment of tooth biomechanical behavior.

## Figures and Tables

**Table 1:** Materials composition.

Resin composite	Manufacturer	Resin matrix	Filler	Batch #
SDR*™* Microhybrid	Dentsply DeTrey	Modified UDMA EBPADMA TEGDMA	Ba-Al-F-B-Si-glass Sr-Al-F-Si-glass (68 wt%., 45 vol%)	1105141
Esthet•X® HD Microhybrid	Dentsply DeTrey	Bis-GMA adduct Bis-EMA adduct TEGDMA	Ba-F-Al-B-Si-glass Nanofiller silica (77 wt%; 60 vol%)	1006292

Adhesive	Manufacturer	Chemical composition	Instructions	Batch #

Prime&Bond®NT*™* 2-step etch and rinse adhesive	Dentsply DeTrey	Di- and trimethacrylate resins PENTA Photoinitiators Stabilizers Nanofillers Acetone	Apply 36% phosphoric acid for 15 seconds; spray and rinse with water for 15 seconds; blot dry conditioned areas; apply adhesive and leave the surface wet for 20 seconds; gently dry for at least 5 seconds; polymerize for 10 seconds; apply a second layer of adhesive	1109001528

Bis-GMA (bisphenol A dimethacrylate); Bis-EMA (bisphenol A polyethylene glycol diether dimethacrylate); UDMA (urethane dimethacrylate); TEGDMA (triethylene glycol dimethacrylate); EBPADMA (ethoxylated bisphenol A dimethacrylate).

**Table 2:** Descriptive statistics analysis.

Time (min)	Resin composite	Deformation (*με*) mean (Std. Deviation)	Minimum (*με*)	Maximum (*με*)	Cuspal deflection (*μ*m) mean (Std. Deviation)
0.5 min	SDR*™*	−646.3 (218.5)	−457.3	−1195.5	−2.5 (0.8)
	Esthet• X® HD	−1299.5 (190.5)	−1077.9	−1582.0	−5.0 (0.7)

5.5 min	SDR*™*	−2074.1 (137.1)	−1910.5	−2371.7	−8.0 (0.5)
	Esthet• X® HD	−2296.9 (256.5)	−1786.7	−2608.6	−8.9 (1.0)

6 min	SDR*™*	−1166.4 (286.4)	−873.9	−1747.3	−4.5 (1.1)
	Esthet• X® HD	−1751.4 (286.3)	−1194.1	−2095.1	−6.8 (1.1)

10 min	SDR*™*	−2001.2 (179.9)	−1655.2	−2341.5	−7.8 (0.7)
	Esthet• X® HD	−2277.2 (260.6)	−1757.5	−2600.6	−8.8 (1.0)

**Table 3:** Mean differences of deformation for samples restored with Esthet• X® HD and SDR*™*, for each time period (ANCOVA).

Time (min)	Levene's test (*p*)	*F*	*p*	Mean difference (SDR - Esthet X) (*με*)	Std. error difference	Mean difference (SDR - Esthet X) (*μ*m)
0.5	0.751	52.69	<0.01^*∗*^	626.8	86.4	2.4
5.5	0.120	7.15	0.016^*∗*^	255.6	95.6	1.0
6	0.733	21.64	<0.01^*∗*^	601.4	129.3	2.3
10	0.620	7.37	0.015^*∗*^	274.6	101.1	1.1

^*∗*^Statistically significant differences.
